# Integrating Sustainability in Radiology: Challenges and Opportunities from the Perspective of Radiology Professionals in Saudi Arabia

**DOI:** 10.3390/healthcare13233038

**Published:** 2025-11-24

**Authors:** Zuhal Y. Hamd, Tahani O. Alkahtani, Lama Almudaimeegh, Awadia Gareeballah, Mohammad Aljamal, Mohamed Abuzaid, Nada Alhaarbi, Bayan Alqarni, Jood Alnoufal, Shmouk Alanazi, Alaa Aldhahi

**Affiliations:** 1 Department of Radiological Sciences, College of Health and Rehabilitation Sciences, Princess Nourah bint Abdulrahman University, P.O. Box 84428, Riyadh 11671, Saudi Arabia; zyhamd@pnu.edu.sa (Z.Y.H.); toalqahtani@pnu.edu.sa (T.O.A.); nada.alharbi002@hotmail.com (N.A.);; 2 Internal Medicine Department, College of Medicine, Princess Nourah bint Abdulrahman University, Riyadh 11671, Saudi Arabia; 3 Department of Diagnostic Radiology, College of applied medical sciences, Taibah University, Al-Madinah 42353, Saudi Arabia; 4 Department of Medical Imaging, Faculty of Allied Medical Sciences, Arab American University, 13 Zababdeh, Jenin P.O. Box 240, Palestine; mohammad.aljamal@aaup.edu; 5 Medical Diagnostic Imaging Department, College of Health Sciences, University of Sharjah, Sharjah P.O. Box 26666, United Arab Emirates; mabdelfatah@sharjah.ac.ae

**Keywords:** green healthcare, sustainability, radiology, environmental awareness, energy conservation, waste management, healthcare practices, Saudi Arabia

## Abstract

**Background/Objective:** This cross-sectional study assessed the knowledge, attitudes, and practices (KAP) of radiology professionals in Saudi Arabia regarding environmental sustainability. **Methods**: An online survey with 298 participants revealed moderate knowledge and positive attitudes towards sustainability. **Results**: However, a significant gap between awareness and practice was observed. The most common sustainable practice was digital documentation (53.4%), while energy saving and water saving measures were rare, with more than half rarely or never practicing energy saving. The primary barrier to implementation was a lack of awareness and training (50%). The sustainability association was significantly higher among older, female, and hospital-based professionals. **Conclusions:** The findings underscore the urgent need for improved educational initiatives, strong institutional support, and clear policy frameworks to effectively integrate sustainable practices into radiology departments in Saudi Arabia.

## 1. Introduction

Healthcare systems contribute significantly to environmental degradation through energy use, waste generation, and greenhouse gas emissions. Radiology departments are particularly resource-intensive owing to their reliance on sophisticated equipment, chemical processes, specialized materials, and continuous power requirements. In Saudi Arabia, rapid healthcare expansion and investment in radiography and imaging services lead to enhanced patient care but also intensified environmental challenges. Traditional film-based imaging produces chemical waste, while modern modalities like CT and MRI consume large amounts of energy. Additionally, the use of material such as contrast agents produces complex waste material. Saudi Vision 2030 emphasizes sustainability across sectors, including healthcare, prompting increased interest in eco-friendly practices [1].

A growing movement known as green healthcare has emerged to address these concerns. It encompasses policies and practices that reduce waste, improve energy efficiency, and limit pollution while maintaining safety and quality standards. A key metric for assessing environmental performance is the ecological footprint, which measures the biologically productive land and water required to provide the resources consumed and absorb the waste generated by human activities [2]. Reducing the ecological footprint of healthcare institutions is therefore central to achieving environmental sustainability and mitigating the sector’s contribution to climate change. The World Health Organization (WHO) has urged countries to adopt methods that reduce their effect on the environment without compromising their patient care, and this is why it is important to institute a major objective of sustainability in healthcare systems. There will be an immense improvement in healthcare systems impacted by climate change. This transformation is identified to be one of the most significant dangers and human barriers. Health and well-being endangering the aim of universal healthcare (UHC) and climate-related health are a more extensive threat. With greenhouse gas (GHG) emissions continuing to rise, the healthcare system must be redesigned to ensure its citizens continue to receive safe and effective care. The healthcare system can play a leading role in this transformation by implementing a fair, sustainable, and effective approach to health and climate change [3]. Climate change is a phenomenon that threatens the health of the global community through increased respiratory diseases, resulting in food insecurity and malnutrition, and natural disasters that disrupt healthcare systems, underscoring the need for immediate action [4].

Globally, there is a growing body of research assessing sustainability in radiology. Recent surveys in Europe [5], Asia, and Africa [6,7] have consistently identified a pattern: radiology professionals generally have positive attitudes towards environmental sustainability but show a significant gap in translating this awareness into daily practice. A common trend in these studies, regardless of economic context, is the identification of major barriers, including lack of training, insufficient institutional support, and financial constraints. Our study, placed in the unique context of Saudi Arabia’s Vision 2030 [8], is in line with these global findings and confirms that these challenges are widespread. The consistent replication of these results in recent years underscores the urgent, worldwide need for structured interventions in the radiology field.

A recent study by Abuzaid et al. examined the knowledge, attitudes, and practices related to sustainability across five countries. The findings revealed that Saudi Arabia had the lowest average scores for both sustainability knowledge and attitudes, while Ghana scored the highest. This suggests that Saudi participants generally showed lower levels of awareness about sustainability [6]. In addition, a systematic review by Alnutaifi et al. (2025) [1] analyzed 37 studies conducted between 2010 and 2023 that focused on various aspects of sustainable practices within Saudi radiology departments. The review highlighted that staff engagement and education were consistent and crucial factors for achieving success across all areas of sustainability [1].

Furthermore, the Saudi Ministry of Health seeks to achieve advanced standards in environmental safety. Through its electronic services, operations, and transactions, the ministry strives to promote environmental conservation and comprehensive sustainability [6]. There are several steps that the Ministry has undertaken to accomplish. This, like enhancing resources, performance review, and raising awareness among the people by raising awareness. It encourages community building and involvement [9].

As per the efforts taken by the Ministry to advance sustainability, in the field of radiology, a critical subject area, ecologically mindful procedures can significantly reduce the ecological footprint of the sector. Radiology departments consume much energy, and, according to the literature, have a huge amount of waste, and the healthcare sector is the target of environmental degradation [5]. Radiographers form a key component of the healthcare system; however, their contribution to the environment and energy consumption is high. Thus, to implement viable measures to reduce the environmental impact of diagnostic imaging services in most countries, it is important to understand the attitudes of radiographers towards the sustainability of the environment. As is well known, the complexity and energy-intensive nature of radiology are reflected in the wide range of sources and activities that contribute to greenhouse gas emissions. The main source of greenhouse gas emission in the radiology sector is the energy usage of imaging equipment, such as computed tomography (CT) scanners and magnetic resonance imaging (MRI) scanners, and other devices, such as reporting workstations. Medical imaging, interventional radiology, and radiotherapy processes lead to the use of materials that greatly add to environmental destruction, like plastics and gloves. As a result, adopting environmental sustainability in radiology practice is crucial for long-term resource conservation and environmental preservation. Few studies have examined radiographers’ knowledge, attitudes, and practices regarding sustainability, especially in Saudi Arabia, despite the yield’s substantial environmental impact [5,10]. Therefore, the study aims to assess radiographers’ knowledge, attitudes, and practices regarding environmental sustainability in medical imaging in Saudi Arabia and highlights its alignment with national (Vision 2030) and global sustainability goals. It also aims at establishing the difficulties of establishing a radiology department’s sustainability in Saudi Arabia.

## 2. Materials and Methods

### 2.1. Study Design and Sampling

This cross-sectional observational study was conducted among radiology professionals working in governmental and private hospitals across Saudi Arabia from January 2025 to June 2025. The sample size collected based on an unknown population: n0 = (Z)^2^ (*p*) (q)/(e)^2^, where **e** is the desired level of precision (i.e., the margin of error) = 0.05; *p* is the (estimated) proportion of the population which has the attribute in question; q is 1 – *p*; *p* = 0.5; and a 95% confidence level gives Z values of 1.96, resulting in 385 respondents. However, only 298 completely answered the questionnaire, resulting in a response rate of 77.4%.

### 2.2. Ethical Approval

The ethical approval was acquired from the Institutional Review Board (IRB) at King Abullah bin Abdulaziz University Hospital, IRB Log Number: (HA-01-R-104), before participant data was collected in the Kingdom of Saudi Arabia (KSA). The research committee followed the methods used in the study, and the data were managed confidentially and used only for research purposes.

### 2.3. Data Collection and Instruments

The survey questionnaire was adapted from previous research papers [6,10] and modified to fit the context of this study. This questionnaire contains the participants’ demographic data and multiple closed-ended questions to explore sustainability knowledge, attitudes, and practices among radiology professionals in Saudi Arabia. The survey questionnaire was drafted in an online Google survey questionnaire, in softcopy and hardcopy. Prior to data collection, the questionnaire was piloted using reliability analysis, including responses of 30 radiology professionals. The results of the pilot, yielding a Cronbach’s alpha of 0.92, demonstrated an excellent level of internal consistency. The online survey link was disseminated randomly via social media platforms (WhatsApp, Telegram, and X). The data collection phase was initiated in January 2025.

### 2.4. Statistical Analysis

Data were analyzed using IBM SPSS version 27 and the DATAtab (version 23) statistical online calculator. The frequencies and percentages were calculated for the categories data, and then, mean, median, and standard deviations were calculated to describe the 33 items in four domains that were rated on a 5-point Likert scale (1 = strongly disagree, 2 = disagree, 3 = neutral, 4 = agree, and 5 = strongly agree), the mean and median were calculated for each question in four domains (knowledge, awareness, practice, and barriers); then, a weighted average was calculated for each domain and for the overall score, where any score for each question equal to or more than weighted average was considered high, while any score less than weighted average was considered low. Before going one step further with the analysis test, the normality test was performed to assess the normality of the score for a five-point Likert scale-rated question. The results of the Kolmogorov and Smirnov tests found that the level of awareness concerning sustainability was non-normal (*p* < 0.001); as a result, the non-parametric tests were considered. (Mann–Whitney U and Kruskal–Wallis H). The level of awareness of sustainability in healthcare was inspected in terms of socio-demographic factors (age, gender, years of experience, and hospital workplace). A *p*-value ≤ 0.05 was considered statistically significant.

## 3. Results

### 3.1. Demographic Characteristics

**Figure 1:**
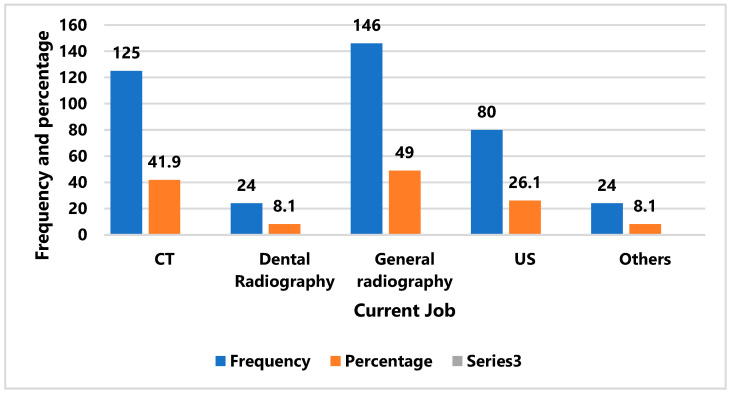
Distribution of current jobs of the respondents. CT: computed Tomography, US: Ultrasound.

Table 1 and Figure 1 provide a summary of the participants’ demographic and vocational characteristics. Most responders were between the ages of 20 and 29 (56.4%), with over half being females (51.3%). Most participants had 1 to 10 years of professional experience (80.2%) and were employed in hospitals (89.9%). Nearly half of those with present jobs were, in general, in radiology and CT fields (49.0% and 41.9%), and very few were in dental radiography or other fields.

### 3.2. Sustainability Focus

The findings from the “focus on sustainability” section are shown in Table 2. Reducing the environmental impact of healthcare operations was the definition of sustainability in healthcare given by nearly half of the participants (48 percent). Water conservation received the least attention (4%), whereas most respondents (68.1%) said “all of the above” when asked about their primary areas of concern. In terms of sustainable practices, water-efficient fixtures were the least popular (8.7%), while digital documentation to cut down on paper use was the most popular (53.4%). Regarding energy saving, “rarely” was the most often chosen response (28.2%), while “monthly” was the least chosen (11.1%). Composting was used infrequently (7%), whereas recycling was the most popular method of disposing of garbage (46%). Lack of knowledge and training was the biggest obstacle to implementing sustainability (50%), while worries about the impact on patient care were just 8.1%. Lastly, just 11.1% of individuals thought of sustainability as a factor when it was convenient, but more than half (51%) thought it was somewhat important but subordinate to patient care.

Table 3 represents the score of knowledge and perception about sustainability in healthcare. The mean scores were 3.08, representing a moderate level of knowledge and perception of sustainability among the respondents, with Q1 (“I understand the concept of sustainability”) and Q7 (“I understand energy conservation in a healthcare setting”) showing the highest levels of agreement, indicating stronger knowledge in these areas. For understanding the concept of sustainability, about 32.2% agree, and 15.1% strongly agree that they understand; furthermore, 32.2 and 16.1% SAG and AG understood the concept of energy conservation in a healthcare setting. In contrast, only 18.1% and 4.0% SAG and AG reported that they were familiar with sustainability in their workplaces, with the lowest mean score (2.64).

The participants’ awareness of sustainability concepts in healthcare is displayed in Table 4. A comparatively high level of awareness is shown by the overall average score of 3.44. Concerning the importance of sustainability in healthcare, most respondents agree and strongly agree (36.9% and 21.1% for AG and SAG), and the mean score was 3.58. Furthermore, with a positive attitude toward the integration of sustainability into all health professional training programs and a motivation to learn more about sustainable healthcare practices, every health professional can promote sustainability (the mean score was 3.52, 3.53, and 3.60, respectively). On the other hand, a negative attitude was noted concerning sustainable practices being essential for improving patient outcomes, sustainability being prioritized at their workplace, and the capacity for the healthcare sector to reduce its environmental impact (the mean score was 3.29, 3.15, and 3.41, respectively).

Table 5 shows the participants’ readiness to practice health sustainability. Based on the results, the highest average value in readiness for adoption for the question “There is a lack of training on sustainable practices and”, with an average of 3.80 (73.9% for SAG and AG regarding the question on the lack of training). Conversely, the lowest-valued question, with an average of 2.66 and 2.62, was “I participate in training/workshops on sustainability in my workplace (only 27.9% mentioned that they were participated training workshop) and I contribute to sustainability feedback processes at work (49% disagreed)”. The average score for adoption and practice was 3.00, indicating moderate readiness to adopt and practice it.

Table 6 assesses the participants’ barriers and obstacles toward sustainability in healthcare. The highest mean score was (3.78), which, with current workload and staff shortages, makes it difficult to prioritize sustainability; about 34.2% SAG and 28.5% agree with it. Concerning the question about the lack of leadership commitment to sustainability, the respondents also demonstrated strong agreement (65.5%); the mean score was 3.71. On the other hand, the question “The benefits of implementing sustainability are not well communicated” yielded the lowest mean value (3.47), and 55% agree with this statement.

**Figure 2:**
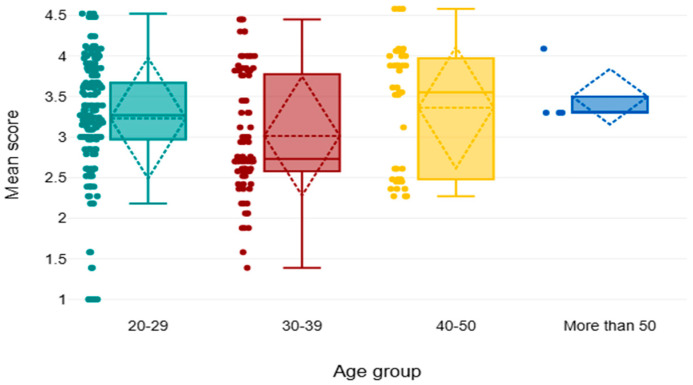
Comparison of the mean score among different age groups.

Table 7 and Figure 2 demonstrate a statistically significant difference in awareness levels across age groups and years of experience regarding age group (Chi^2^ = 10.53, *p* < 0.05). Although the age group of 30–39 had the least awareness, with a mean rank of 124.72, the age group between 40 and >50 years of age (mean rank = 166.37 and 187.13, respectively) exhibited greater awareness. Furthermore, the result shows a significant increase in the level of perception and attitude concerning sustainability, with employees who had worked >20 years reporting the highest mean rank = 204.67, with a *p*-value < 0.05, chi^2^ (6.68).

**Figure 3:**
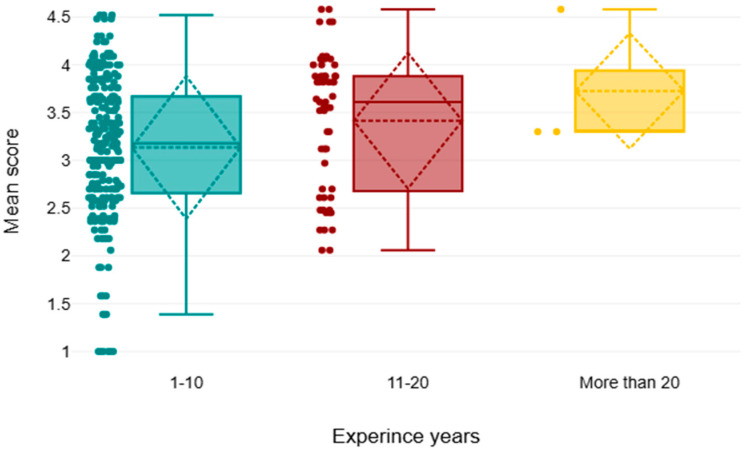
Comparison of the mean of different years of experience.

The result showed that there was a significant difference between the categories of years of experience with respect to the knowledge of sustainability in radiology, where *p* = 0.002; the score of knowledge increased as the year of experience increased. The Dunn–Bonferroni test showed that the pairwise group comparison of 1–10–11–20 significantly differs, with a *p*-value of less than 0.05. Considering the difference in degree of awareness and in different experiences, the results showed a Chi-square value of 8.82 (*p*-value of 0.012), indicating that there is a statistically significant difference in attitude across the three groups being compared; the post hoc test shows significant differences in attitude across pair 1–10–11–20, with an adj. *p*-value of 0.01. Insignificant differences were observed in practice and barrier score in different years of experience (*p*-values of 0.212 and 0.227, respectively); see Table 8 and Figure 3.

**Figure 4:**
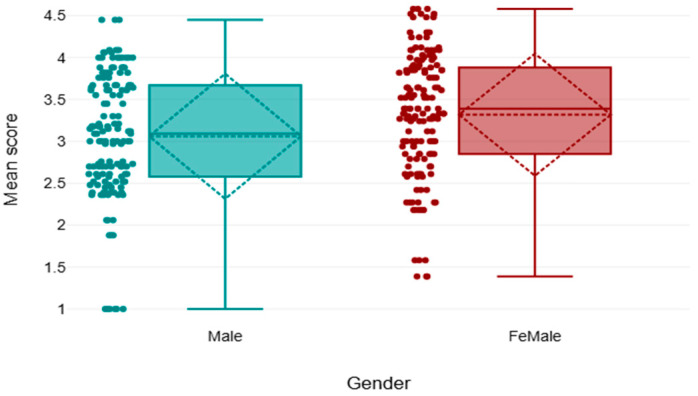
Comparison of mean scores between both genders is weak.

The results demonstrated a significant difference in the overall sustainability scores between male and female participants, and across areas of work. The female gender demonstrated a greater mean rank than the male (mean rank = 163.99, U = 8875.5, and *p* < 0.05) (Figure 4).

**Figure 5:**
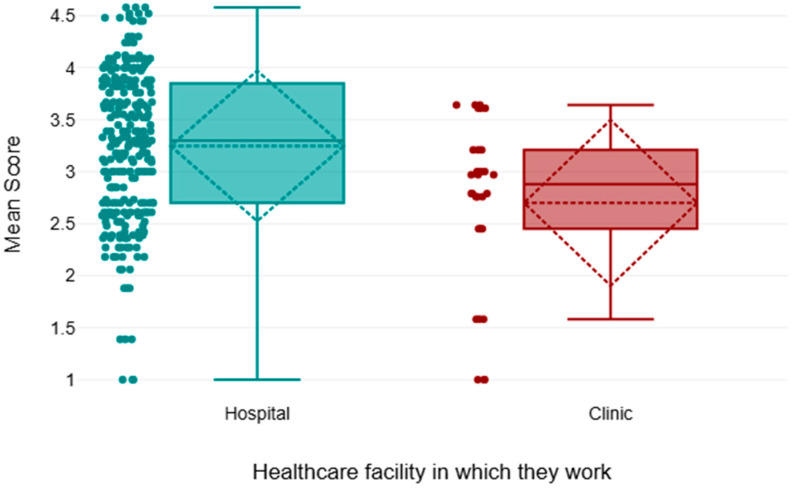
Comparison of mean scores in both healthcare facilities where they work.

Employees working in hospital settings demonstrated a higher mean rank (154.89) in sustainability awareness compared to those in clinics, with a statistically significant difference (*p* < 0.05); see Table 9 and Figure 5.

## 4. Discussion

The purpose of this research was to evaluate Saudi Arabian radiology professionals’ attitudes, knowledge, and behaviors around sustainability. The research showed that radiographers were keen to incorporate sustainability into their everyday work and reported having a positive opinion of it. However, the primary obstacles to prioritizing sustainability include a lack of leadership commitment to sustainability, present workload and personnel limitations, and a lack of training.

### 4.1. Knowledge and Perception

The study demonstrated that radiography professionals generally have a positive perception of sustainability in healthcare, aligning with those reported in the European study [6]. Yet it also reveals critical gaps that require attention. In both studies, participants recognized the importance of environmental sustainability, and in the present survey, 48% of the participants defined the concept of sustainability in healthcare as the focus to reduce the impact on the environment. Further, a majority (68.1%) of the respondents in this survey identified waste management, water conservation, and energy efficiency to be important components of sustainable healthcare, which is in line with the multifaceted perceptions of the European sample [6]. Nevertheless, the differences are observed when considering more daily sustainable activities: despite (53.4%) of the participants stating that they used a digital documentation to reduce paper consumption which aligns with its application in the comparing study, other greener practices, such as water-efficient mixtures and light-saving bulbs, were considerably underused (13.8% and 8.7%, respectively), which may indicate an institutional or infrastructure constraint. The energy-saving measures of 22.1% of the participants who actively worked to cut energy consumption daily is relatively low participation in energy saving measures when compared to the high levels of participation in the energy saving measures that are already high in the reference study. This pattern of not practicing such activities widely is further reiterated by the high percentage of participants who hardly (28.2%) or never (26.8%) participate in such activities. In addition, the degree of knowledge about sustainable waste disposal methods was also different; the most frequently used strategy was recycling and composting. This trend was also replicated, albeit slightly better in the European context. The notable similar results in one of the key findings of the two studies are that there is a considerable barrier that arises due to a lack of knowledge and training (50% in this study), the constraints of the budget, and managerial support. This demonstrates that it is systemic problems that hinder sustainability and not individual resistance. Finally, sustainability, although only 23.2% of them believed it to be one of the main clinical concerns, was perceived by more than half of the participants as an addition to patient care. This is in line with data from Europe, where clinical outcomes were still given top priority. When taken as a whole, these findings show that healthcare professionals recognize the value of sustainability, but they also show that to enable effective application in clinical practice, specific education, policy formulation, and infrastructure support are required. In comparison to this study, one study conducted in Zimbabwe and Zambia found that most radiographers were aware of the concept of sustainability. The radiography educational curriculum was identified as having insufficient emphasis on sustainability (44.44%). More than half of the radiographers indicated a lack of sustainable practices in their departments. The most reported barriers to sustainability are a lack of priority for sustainability from leadership and organizations, a lack of incentives for sustainability, and a lack of collaboration between suppliers and consumers on ways to improve diagnosis, patient safety, and sustainability [7]. Another study agrees with this study and reports that most radiographers comprehended the concept of sustainability in radiography; nevertheless, they expressed concerns regarding the environmental impact of radiographic practices and sought additional training and financial assistance to mitigate these effects [11]. Another study conducted in Europe mentioned that most participants thought that environmental sustainability in healthcare was vital. And in agreement with this study, most of the respondents reported they were either ignorant of or did not have environmental sustainability practices in place at work [5]. Rawashdeh et al. reported that the majority of those surveyed contemplate that sustainability practices need to be improved. The most popular methods for reducing emissions and energy usage were energy-efficient heating systems (32%), real-time power monitoring tools (41%), and low-energy lighting (60%). Respondents’ worries about sustainability are noteworthy: among the constraints highlighted, time and lack of leadership are the most common problems [4].

A moderate level of knowledge and perception was noticed about sustainability in healthcare among the radiographers; most of the respondents reported that they understood the concept of sustainability, were aware of the impact of sustainability practices on patient care, and were aware of the environmental impact of waste from healthcare facilities, understanding the vitality of energy conservation in a healthcare setting. On the other hand, a considerable number of them reported “low familiarity with my workplace’s sustainability practices”, which indicates the lack of institutional knowledge or communication. These findings are consistent with Abuzaid et al., who found that while the majority of the radiologists were aware of the general concept of sustainability, familiarity with workplace sustainability policies was lower, and many respondents also acknowledged the environmental impact of healthcare waste and understood the importance of energy conservation within healthcare [10]. Therefore, both studies demonstrate the same trend: while general awareness is present, practical knowledge and workplace engagement remain limited, indicating a need for stronger institutional communication and more effective implementation of sustainability policies.

### 4.2. Attitude

The findings of this research show that the participants have a mostly positive attitude towards the issue of health sustainability, with a weighted average score of 3.44. It is worth noting that question 13, which is “Every health professional can promote sustainability”, was rated with the highest level of agreement (mean = 3.6), and, therefore, it can be concluded that there is a profound belief in what an individual healthcare professional can do to help in the development of sustainable practices. The fact that sustainability is commonly recognized as a key factor of healthcare education and practice is also supported by the high ratings of question 8, which emphasizes its importance in healthcare (mean = 3.58) and its integration in professional training in question 11 (mean = 3.52). But question 10, “Sustainability is prioritized at my workplace” was the least in terms of mean score (3.15), indicating perceived inconsistency between institutional action and cognizance. This gap means that, despite the awareness by healthcare professionals of the need to be sustainable, more organizational and systemic interventions may be required to translate the beliefs into working processes.

### 4.3. Practice

The most significant results of Table 5 were the following: In total, (38.3) of participants agree and adhere to sustainability principles in the workplace. A total of (46%) attempt to minimize waste in their daily job activities. Altogether, (36.9%) encourage others to adopt sustainable practices at work. In total, (35.2%) of participants agree with using resources responsibly to reduce environmental impact. A total of (43%) support initiatives aimed at reducing energy consumption. Overall, (32.8%) try to implement sustainable practices in their daily activities. A total of (60.7%) regularly evaluate the effectiveness of sustainability practices. A total of (48%) follow appropriate waste disposal procedures. In all, (37.3%) agree to consider the environmental impact of their clinical decisions. This is evidence that there are people who care about sustainability, and we must strive to increase this percentage by encouraging healthcare workers to follow sustainability guidelines. However, there are (49%) who do not participate in training/workshops on sustainability in healthcare when available, (36.9%) who do not make suggestions for improving sustainability, (39.9%) do not seek out sustainable product alternatives for use in my clinical practice, (49%) do not contribute to sustainability feedback processes at work, and (45%) do not actively engage in discussions about sustainability issues at work. The fact that 73.9% of participants agreed that there is insufficient training on sustainable practices is one of the significant findings in Table 5 and the study [6]. Like our study, others report that there is a need for training results, and study [6] reported that there is insufficient training information (36%). This is enough proof that training in sustainable practices is especially necessary. Additionally, it shows that some aspire to protect the environment. Finally. The results of Table 5 indicate that the total weighted average was 3.00, and this indicates that most participants demonstrated low levels of awareness regarding sustainability practices in a healthy workplace. This general pattern reflects a clear gap between the theoretical concepts of sustainability and their practical application in daily practice, calling for institutional intervention to improve knowledge, change behaviors, and provide appropriate tools to facilitate the adoption of these practices.

One discussion paper, like our study, reported that the radiographers must comprehend the factors contributing to the carbon footprint and collaborate with sponsors to curtail these impacts, and it is also necessary for radiographers to engage in sustainability education programs and research to enhance their knowledge and to practice sustainably [12]. Abdul Razak et al. agree with our findings, highlighting the requirement for sustainable practices in radiology departments and signifying that integrating sustainability training into professional development and the radiography curriculum could improve radiographers’ environmental awareness and implementation [13]. Debnath et al. also mention that most respondents reported that education and training could enhance green and sustainable practice in radiography [14].

### 4.4. Barriers

The respondents demonstrated familiarity with barriers to sustainability practice in radiology; a large portion of the participants, 89 (29.9%), agreed that there is not enough financial support to implement sustainable practices. Most participants agreed that the current workload and staff shortages make it difficult to prioritize sustainability (34.2%). This percentage indicates the importance of restructuring the workload and providing sufficient human resources to activate sustainability in the healthcare sector, especially in the radiology department. In total, (40.3%) of participants agreed that there is a lack of leadership commitment to sustainability. The participants thought that the benefits of implementing sustainability had not been properly communicated, where (41.9%) indicated a gap in communication and education about the benefits of sustainability, and these factors decreased the enthusiasm of the employees and/or stakeholders. Based on these results, we concluded that the major challenges to implementing sustainability extend to include weaknesses in the administrative and organizational aspects. Therefore, we recommend the need to develop integrated subscriptions that include financial support, awareness, and development for employees, and strive to make the work environment more aware and sustainable. Study [8] agreed with the results of our study, as it stated that the current study identified the main barriers to sustainability, including lack of training (39.2%), financial constraints (44.7%), and insufficient administrative support (39.2%).

The results showed that there was a statistically significant difference in awareness levels across age groups, with those who were more than 50 years old demonstrating the highest level of awareness, and 30–39 years old demonstrating the lowest level of awareness. These findings show that there is a tendency to be increasingly aware of sustainability as age increases. The results show an increase in sustainability awareness and commitment as the number of years of professional experience increases. Specifically, participants with more than 20 years of experience had the highest mean rank (204.67), followed by those with 11–20 years (173.09), and finally those with 1–10 years of experience (143.28). This was a statistically significant trend with the chi-square value indicating this (χ^2^ = 6.68, *p* = 0.035), indicating that the differences in the sustainability scores between the experience groups were not by chance. Although the sample of the group of over 20 years was very small (n = 3), their higher scores could be attributed to the fact that they are more familiar with the institutions and adhering to sustainability principles, and these scores might be more related to long-term experience. These results also indicate that the more people become familiar with healthcare systems and professional duties, the more they might familiarize themselves with sustainability and be committed to it. This may be because of experience gained over time, more understanding of the impacts of organizations and the environment, or more experience with the long-term effects of unsustainable practices.

Furthermore, this study identified a significant gender disparity in sustainability awareness, knowledge, practice, and barriers, with female radiographers scoring higher than males. Perhaps women are more inclined toward preventive and socially responsible behavior, something that lies within their historically stronger role in caregiving and community service-like responsibilities. The explanation is consistent with current evidence in radiology. For example, in “Environmental Sustainability in Radiography”, as well as “Green Radiography—Examining Perspectives”, both highlighted that female professionals demonstrated greater motivation towards applying environmentally sustainable practices, above all, energy saving and waste minimization. In addition, “Bridging the Gap in Sustainable Radiography” revealed that female staff perceive sustainability as integral to patient care and not as an additional responsibility. Contrary to this, male participants may appreciate technical efficiency at the expense of environmental concerns, something which may partly describe the observed difference.

The difference in scores between the staff based in the hospital could be due to several contextual factors. Hospitals are usually bigger compared to clinics, have more elaborate infrastructure, and demand more resources, including energy, water, and materials. Such a level of operations can make healthcare delivery more direct to the hospital employees, thus increasing their understanding and interest in sustainability practices. In addition, bigger institutions tend to meet stricter environmental rules and sustainability efforts, and training programs may also lead to increased institutional focus on sustainability. By comparison, the clinical environments might not have the same institutional pressures and resources to encourage sustainability, which might be the reason why the clinic staff scores have decreased. Hospitals, which, in most cases, have more complex administrative systems, environmental practices, and financial support of sustainability programs, may create more awareness and practice of environmentally friendly practices among staff. These results are very consistent with the results of the study [11]; in their case, the participants were 441 radiographers; most of them (71.5%) were employed in hospitals, and they were more likely to report knowing about sustainability concepts and practicing eco-friendly behavior, including preventing the use of paper and recycling. On the other hand, those in a clinical setting were not likely to pursue such practices, where limited training and a deficiency of leadership commitment were cited as the reasons. The researchers concluded that institutional support, particularly in hospital settings, is a major contributor to sustainability awareness and practice. Collectively, these findings illustrate how workplace infrastructure and policy can have an impact on sustainability behavior in radiological practice. Targeted strategies, such as increasing sustainability training and support in clinics, may help bridge the gap and promote more consistent environmental practices across healthcare settings. The Saudi Arabian research paper in this study is generally comparable to that of other countries. Like in Europe, radiology personnel indicated moderate knowledge and positive attitudes towards sustainability, but with little identification of workplace-specific activities [6]. Awareness is weaker, and there is little more than primary recycling practiced in Africa due to a lack of resources; however, in Asia, awareness is increasing, particularly in terms of equipment efficiency, but it is inconsistent. In practice, Saudi radiographers reported high rates of digital documentation and recycling, but low use of infrastructure interventions, such as energy-efficient lighting and water-saving measures. This pattern is less advanced than in Europe but superior to that in most African settings [4,10]. Saudi Arabian barriers—lack of training availability, financial constraints, and inadequate leadership support—are mirrored in the documented ones elsewhere, although more severe in Africa and gradually in Asia [8]. The demographic trends also indicated more awareness among longer-serving, older, and hospital-based staff, as would be expected from European data and no doubt shaped by their institutional structure and professional maturity [6]. The general trend is an indication of an actual gap between the theoretical knowledge of sustainability and the real practice in daily life, which requires institutional intervention to enhance knowledge and change behavior, and offer the appropriate tools that would help adopt these practices. Moreover, institutional problems, including a lack of financial resources, excessive workloads, and the absence of leadership support, also contribute to it. Thus, it is necessary to pay attention to solving these problems to make the practical implementation of sustainability in healthcare better. Furthermore, our study results and participant responses align with Sustainable Development Goals, SDG 3: Good Health; SDG 12: Responsible Consumption; and SDG 13: Climate Action, with participants reporting sustainability practices such as the use of reusable materials, water-efficient mixtures, and energy-efficient lighting, all of which encourage responsible resource consumption. This leads to reduced carbon emissions associated with energy production and consumption, which supports Goal 12. The practices also assist Sustainable Development Goal 13. These practices also enhance health and safety in the healthcare facilities by reducing the exposure to harmful chemicals and waste, which is a part of Sustainable Development Goal 3 [15].

In conclusion, the study demonstrated that there are several barriers that put the participants in a position that prevents their adoption of sustainable healthcare practices. These barriers are poor administrative support, equipment, a deficiency of knowledge and training, and finance. These findings mean that despite the current objectives and programs of the Saudi Ministry of Health, the training programs need should be enhanced; the administrative and institutional support should be advanced to a higher level. Overall, these findings highlight the need for further research focusing on larger and more diverse samples, interventional designs, and direct evaluation of training and policy initiatives to accelerate the integration of sustainability principles into Saudi healthcare practice.

## 5. Conclusions

The study concluded that the radiology professionals in Saudi Arabia demonstrated moderate knowledge, perception, and attitude. Furthermore, lacking the practice of sustainability in radiography, they were aware of sustainability and environmental conservation but were deficient in practical implementation; the biggest obstacles to implementing sustainability practices in healthcare are the deficiency in training, lack of institutional leadership, and workload. With significant differences in the mean score of sustainability knowledge, perception, attitude, and barriers across different genders, age groups, areas of work, and years of experience, the highest score of knowledge and practice was noted in females, older age groups, governmental hospital workers, and those with more years of experience. The only way to close this gap is through intensive educational activities, institutional support, and policy formulation. Thus, there is an urgent need to introduce policy-based interventions and specific training programs to integrate sustainability into the everyday practice of radiology. Embedding sustainability into radiology curricula and hospital accreditation standards will accelerate the transition toward environmentally responsible healthcare.

### 5.1. Practical Implications

Findings highlight several actionable strategies for healthcare institutions. Introducing energy-saving technologies like optimized CT parameters, low-power MRI scanners, and automatic standby systems can substantially reduce energy consumption. Implementing structured waste management, including recycling and safe disposal of contrast agents, further decreases environmental harm.

### 5.2. Theoretical Implications

This study contributes to the emerging theoretical understanding of sustainability behavior in healthcare. It illustrates how individual knowledge and institutional structures jointly influence environmental practices. The results support integrating behavioral and organizational theories—such as the Theory of Planned Behavior and Institutional Theory—to explain how awareness, motivation, and leadership shape sustainable actions. The findings also call for multidisciplinary theoretical frameworks combining healthcare management, environmental science, and behavioral psychology to guide sustainability adoption in radiology and other high-impact medical fields.

### 5.3. Recommendations

It is important to provide a regulation that entails the adoption of sustainable practices by the radiology departments and implement comprehensive awareness initiatives to educate all radiographic specialists about the vital role sustainable practices play in maintaining the healthcare system and the environment, and to encourage further research into this topic.

### 5.4. Directions for Future Research

Expanding on these findings, future studies should

- Use larger, more stratified data sets to improve representation across Saudi healthcare professions.
- Tracking changes in behavior and the environment around us by periodic assessments of training or technological improvements.
- Examining different countries’ strategies to identify the sustainability models that perform effectively. Investigating AI, Internet of Things (IoT), and automation technologies can further optimize radiology’s energy use and waste handling.
- Exploring behavioral interventions such as feedback systems or gamification to motivate sustainable actions among radiographers.

### 5.5. Interdisciplinary Collaboration

Sustainability in radiology demands a truly interdisciplinary approach. Environmental scientists can develop tools for measuring imaging-related emissions; engineers can design energy-efficient equipment; behavioral scientists can create strategies to change staff habits; and healthcare administrators can embed sustainability metrics in institutional policies. Such cross-sectoral collaboration is vital for creating practical, scalable solutions that balance clinical performance with ecological responsibility.

### 5.6. Role of Technology

Technological innovation is central to sustainable radiology. Artificial intelligence (AI) can optimize scan parameters, minimize repeated exposure, and reduce energy consumption. Next-generation scanners are being designed with reduced power requirements, and cloud-based image archiving lessens reliance on physical storage. Tele-radiology services also cut patient travel, reducing carbon emissions. Policymakers should prioritize investment in such digital solutions and ensure interoperability standards to maximize both environmental and clinical efficiency.

### 5.7. Education Reform

Bridging the gap between awareness and action requires targeted education. Radiography curricula should integrate modules on green healthcare, including energy management, waste control, and environmental impact assessment. Continuing professional development (CPD) programs should reinforce these principles through workshops and certifications. Interprofessional learning initiatives that bring together healthcare practitioners, environmental experts, and administrators can promote shared responsibility and systems thinking. Embedding sustainability in education ensures that future radiographers are both technically proficient and environmentally accountable.

### 5.8. Limitation

One limitation of this study was the small sample size, which may have decreased the accuracy of the analysis, suggesting an increased number of samples to obtain more accurate results. The final limitation was that the survey was distributed randomly via social media platforms and professional networks, which may have introduced selection bias. Furthermore, inequality of distribution of participants in different categories of demographics (i.e., gender and area of work) may lead to correlation bias.

## Figures and Tables

**Table 1:** Demographic characteristics.

Demographic Characteristics		Frequency	Percent
Gender	Male	145	48.7
	Female	153	51.3
Age group	20–29	168	56.4
	30–39	84	28.2
	40–50	42	14.1
	More than 50	4	1.3
Healthcare facility	Hospital	268	89.9
	Clinic	30	10.1
Experiences	1–10	239	80.2
	11–20	56	18.8
	More than 20	3	1.0
	Total	298	100.0

**Table 2:** Demonstration of the results of the sustainability focus.

Question	Answer	n (%)
Which of the following best describes sustainability in healthcare?	Reducing the environmental impact of healthcare operations	143 (48.0)
	Increasing the financial profits of healthcare facilities	51 (17.1)
	Enhancing patient comfort regardless of environmental cost	60 (20.1)
	None of the above	44 (14.8)
What are the key areas of focus in sustainable healthcare?	Water conservation	12 (4.0)
	Energy efficiency	39 (13.1)
	Waste management	44 (14.8)
	All the above	203 (68.1)
Which sustainable practices are incorporated into your daily routine? (Select all that apply)	Digital documentation to reduce paper use	159 (53.4)
	Energy-efficient lighting	41 (13.8)
	Water-saving fixtures	26 (8.7)
	Using reusable materials	72 (24.2)
How often do you engage in practices to reduce energy consumption at your workplace?	Daily	66 (22.1)
	Weekly	35 (11.7)
	Monthly	33 (11.1)
	Rarely	84 (28.2)
	Never	80 (26.8)
What type of waste disposal methods are you familiar with in your healthcare setting?	Chemical treatment	51 (17.1)
	Incineration	89 (29.9)
	Recycling	137 (46.0)
	Composting	21 (7.0)
What is the biggest barrier to implementing sustainable practices in healthcare?	Insufficient managerial support	54 (18.1)
	Lack of awareness and training	149 (50.0)
	Financial constraints	71 (23.8)
	Perceived impact on patient care	24 (8.1)
How do you prioritize sustainability in your clinical decisions?	It’s a primary consideration	69 (23.2)
	It’s of some importance, but secondary to patient care	152 (51.0)
	I consider it when it’s convenient	33 (11.1)
	I do not consider it at all	44 (14.8)

**Table 3:** Demonstration of knowledge and perception of sustainability concepts in healthcare.

Knowledge Questions	Responses (Frequency and %)					Mean	Median	Std. Dev	Decision
	SDA	DA	Neutral	AG	SAG				
Q1, I understand the concept of sustainability	27 (9.1%)	38 (12.7%)	92 (30.9%)	96 (32.2%)	45 (15.1%)	3.32	3	1.15	High
Q2, I am aware of the impact of sustainability practices on patient care	21 (7.5%)	57 (19.0%)	113 (37.8%)	86 (28.2%)	21 (7.5%)	3.1	3	1.02	High
Q3, I am familiar with my workplace’s sustainability	54 (18.1%)	77 (25.8%)	101 (33.9%)	54 (18.1%)	12 (4.0%)	2.64	3	1.1	Low
Q4, I can identify the benefits of implementing sustainable practices in healthcare settings	30 (10.1%)	50 (16.8%)	89 (29.9%)	93 (31.2%)	36 (12.1%)	3.18	3	1.16	High
Q5, I regularly update my knowledge on sustainability	36 (12.1%)	80 (26.8%)	92 (30.9%)	54 (18.1%)	36 (12.1%)	2.91	3	1.19	Low
Q6, I am aware of the environmental impact of waste from healthcare facilities	33 (11.1%)	50 (16.8%)	90 (30.2%)	80 (26.8%)	45 (15.1%)	3.18	3	1.21	High
Q7, I understand energy conservation in a healthcare setting	30 (10.1%)	39 (13.1%)	91 (30.5%)	90 (30.2%)	48 (16.1%)	3.29	3	1.18	High
Weighted average: 3.09									

**Table 4:** Demonstration of awareness and attitudes towards health sustainability.

Attitude Questions	Responses (Frequency and %)					Mean	Median	Std. Dev	Decision
	SDA	DA	Neutral	AG	SAG				
Q8, Sustainability is important in healthcare	15 (5.0%)	33 (11.1%)	77 (25.8%)	110 (36.9%)	63 (21.1%)	3.58	4	1.09	High
Q9, Sustainable practices are essential for improving patient outcomes.	30 (10.1%)	33 (11.1%)	100 (33.6%)	90 (30.2%)	45 (15.1%)	3.29	3	1.16	Low
Q10, Sustainability is prioritized at my workplace.	18 (6.0%)	71 (23.8%)	104 (34.9%)	57 (19.1%)	48 (16.1%)	3.15	3	1.14	Low
Q11, Sustainability should be integrated into all health professional training programs.	12 (4.0%)	32 (107%)	99 (33.2%)	95 (31.9%)	60 (20.1%)	3.52	4	1.1	High
Q12, I am motivated to learn more about sustainable healthcare practices.	12 (4.0%)	48 (16.1%)	68 (22.8%)	113 (37.9%)	57 (19.1%)	3.53	4	1.05	High
Q13, Every health professional can promote sustainability.	12 (4.0%)	27 (9.1%)	94 (31.5%)	99 (33.2%)	66 (22.1%)	3.6	4	1.05	High
Q14, The Healthcare sector can reduce its environmental impact.	24 (8.1%)	39 (13.1%)	77 (25.8%)	107 (35.9%)	51 (17.1%)	3.41	4	1.15	High
Weighted average: 3.44									

**Table 5:** Demonstration of the participants’ readiness for adoption and practicing sustainability in healthcare.

Questions for Practice	Responses (Frequency and %)					Mean	Median	Std. Dev	Decision
	SDA	DA	Neutral	AG	SAG				
Q15, I follow sustainability guidelines at work.	27 (10.8%)	71 (23.8%)	86 (28.9%)	90 (30.2%)	24 (8.1%)	3.04	3	1.11	Low
Q16, I try to reduce waste in my daily work activities.	27 (10.8%)	62 (20.8%)	72 (24.2%)	90 (30.2%)	47 (15.8%)	3.23	3	1.21	High
Q17, I encourage others to adopt sustainable practices at work.	48 (16.1%)	68 (22.8%)	72 (24.2%)	89 (29.9%)	21 (7.0%)	2.89	3	1.2	Low
Q18, I participate in training/workshops on sustainability in healthcare when available.	65 (21.8%)	81 (27.2%)	69 (23.1%)	56 (18.8%)	27 (9.1%)	2.66	3	1.26	Low
Q19, I use resources responsibly to minimize environmental impact	24 (8.1%)	59 (19.8%)	110 (36.9%)	72 (24.1%)	33 (11.1%)	3.03	3	1.15	Low
Q20, I consider the environmental impact of my clinical decisions.	36 (12.1%)	56 (18.8%)	95 (31.9%)	84 (28.2%)	27 (9.1%)	3.1	3	1.09	Low
Q21, I support initiatives aimed at reducing energy consumption.	36 (12.1%)	35 (11.7%)	99 (33.2%)	98 (32.9%)	30 (10.1%)	3.17	3	1.14	Low
Q22, I make suggestions for improving sustainability practices at work.	33 (11.1%)	77 (25.8%)	99 (33.2%)	63 (21.1%)	26 (8.7%)	2.91	3	1.12	Low
Q23, I follow proper disposal procedures for different types of waste.	24 (8.1%)	60 (20.1%)	71 (23.8%)	92 (30.9%)	51 (17.1%)	3.29	3	1.2	High
Q24 I actively engage in discussions about sustainability issues at work.	39 (13.1%)	95 (31.9%)	89 (29.9%)	48 (16.1%)	27 (9.1%)	2.76	3	1.15	Low
Q25, I seek out sustainable product alternatives for use in my clinical practice.	45 (15.1%)	74 (24.8%)	93 (31.2%)	60 (20.2%)	26 (8.7%)	2.83	3	1.17	Low
Q26, I try to implement sustainable practices in my daily life activities.	30 (10.1%)	68 (22.8%)	102 (34.2%)	75 (25.16%)	23 (7.7%)	2.98	3	1.09	Low
Q27, I contribute to sustainability feedback processes at work.	42 (14.1%)	104 (34.9%)	95 (31.9%)	39 (13.1%)	18 (6.0%)	2.62	3	1.07	Low
Q 28 I evaluate the effectiveness of my sustainability practices regularly.	15 (5.0%)	36 (12.0%)	66 (22.1%)	118 (39.6%)	63 (21.1%)	2.76	3	1.11	Low
Q29 There is a lack of training on sustainable practices.	15 (5.0%)	24 (8.1%)	39 (13.1%)	148 (49.7%)	72 (24.2%)	3.8	4	1.05	High
Weighted average: 3.00									

**Table 6:** Demonstration of barriers related to sustainability in healthcare.

Barriers Questions	Responses (Frequency and %)					Mean	Median	STD	Decision
	SDA	DA	Neutral	AG	SAG				
Q30 There is insufficient financial support to implement sustainable practices.	39 (13.1%)	89 (29.9%)	95 (31.9%)	54 (18.1%)	21 (7.0%)	3.6	4	1.1	Low
Q31 Current workload and staff shortages make it difficult to prioritize sustainability	15 (5.0%)	27 (9.1%)	69 (23.2%)	85 (28.5%)	102 (34.2%)	3.78	4	1.16	High
Q32 There is a lack of leadership commitment to sustainability.	18 (6.0%)	21 (7.0%)	64 (21.5%)	120 (40.3%)	75 (25.2%)	3.71	4	1.1	High
Q33 The benefits of implementing sustainability are not well communicated.	18 (6.0%)	27 (9.1%)	89 (29.9%)	125 (41.9%)	39 (13.1%)	3.47	4	1.03	Low
Weighted average 3.64									

**Table 7:** Comparison of the overall mean rank and median among different age groups.

Categories	Domains		n	Median	Mean Rank	Chi^2^	*p*
Age groups	Knowledge	20–29	168	3.07	146.18	6.28	0.099
		30–39	84	2.86	140.84		
		40–50	42	3.43	175.19		
		More than 50	4	3.57	201		
	Attitude	20–29	168	3.71	157.32	9.64	0.022
		30–39	84	3	125.37		
		40–50	42	4	166.42		
		More than 50	4	3.43	150.38		
	Practice	20–29	168	3.07	163.61	20.44	<0.001
		30–39	84	2.53	113.96		
		40–50	42	3.27	160.07		
		More than 50	4	3.07	192		
	Barriers	20–29	168	3.5	136.56	10.05	0.018
		30–39	84	4	171.46		
		40–50	42	4	158.96		
		More than 50	4	3.5	132.38		
	Overall	20–29	168	3.27	156.78	10.53	0.015 *
		30–39	84	2.73	124.71		
		40–50	42	3.55	166.37		
		>50	4	3.3	187.13		
Total			298	3.26			

* Note: For attitude, the Adj. *p* with Bonferroni correction = 0.033 for pairwise comparison of 20–29 and 30–39 years. Adj. *p*: Values adjusted with Bonferroni correction < 0.001 for pairwise comparison of 20–29 and 30–39 years in the practice score, whereas 30–39–40–50 = 0.028; Adj. *p*: Values adjusted with Bonferroni correction for pairwise comparison for 20–29 and 30–39 years regarding the barrier score = 0.014; Adj. *p*: Values adjusted with Bonferroni correction for pairwise comparison for overall score of knowledge, attitude, practice, and barriers = 0.032 for pairs 20–29 and 30–39 years.

**Table 8:** Comparison of the overall mean rank and median among different years of experience.

Domains		Experience/Years	Numbers	Median	Mean Rank	Chi^2^	*p* Value
Knowledge	Experiences	1–10	239	3.00	140.95	12.78	0.002 *
		11–20	56	3.57	181.71		
		>20	3	3.57	229.00		
Awareness		1–10	239	3.57	142.16	8.82	0.012 *
		11–20	56	4.14	179.67		
		>20	3	3.43	170.83		
Practice		1–10	239	3.00	145.63	3.10	0.212
		11–20	56	3.27	163.05		
		>20	3	3.07	204.67		
Barriers		1–10	239	3.75	145.51	2.97	0.227
		11–20	56	4.00	167.16		
		>20	3	3.5	137.50		
Overall		1–10	239	3.18	143.28	6.68	0.035 *
		11–20	56	3.61	173.09		
		>20	3	3.3	204.67		
Total			298	3.26			

* Note: For knowledge, the Adj. *p*: with Bonferroni correction = 0.004 for pairwise comparison of 1–10 and 11–20 years; for attitude, it was 0.01 for this same pair, 1–10 and 11–20 years.

**Table 9:** Comparison of the mean rank and median by gender and areas of work.

Categories			n	Mean Rank	U	Z	*p*	r
Gender	Knowledge	Male	145	139.80	9685.50	−1.90	0.059	0.11
		Females	153	158.70				
	Attitude	Male	145	130.89	8394.00	−3.64	<0.001	0.21
		Females	153	167.14				
	Practice	Male	145	134.47	8913.00	−2.93	0.003	0.17
		Females	153	163.75				
	Barriers	Male	145	150.48	10,950.00	−0.19	0.849	0.01
		Females	153	148.57				
	Overall	Male	145	134.21	8875.5	−2.98	0.003	0.17
		Female	153	163.99				
Area of work	Knowledge	Hospital	268	154.76	2611.50	−3.15	0.002	0.18
		Clinic	30	102.55				
	Attitude	Hospital	268	154.10	2787.00	−2.76	0.006	0.16
		Clinic	30	108.40				
	Practice	Hospital	268	154.26	2743.50	−2.85	0.004	0.17
		Clinic	30	106.95				
	Barriers	Hospital	268	153.04	3070.50	−2.14	0.034	0.12
		Clinic	30	117.85				
	Overall	Hospital	268	154.89	2575.5	−3.23	0.001	0.19
		Clinic	30	101.35				
Total			298					

## Data Availability

Upon request, all related case data will be provided by the main author.

## Abbreviations

The following abbreviations are used in this manuscript:

MRI	Magnetic Resonance Imaging
CT	Computed Tomography
US	Ultrasound
PNU	Princess Nourah bint Abdulrahman University
IRB	Institutional Review Board
SD	Standard Deviation
GBCA	Good Gadolinium-Based Contrast Media
GHG	Greenhouse Gas
ACR	American College of Radiology
SDGs	Sustainable Development Goals
WHO	World Health Organization
UHC	Universal Healthcare
SDA	Strongly Disagree
DA	Disagree
AG	Agree
SAG	Strongly Agree
